# Plummer–Vinson syndrome: a rare occurrence in paediatrics

**DOI:** 10.1186/s12887-024-04750-x

**Published:** 2024-04-27

**Authors:** Wasif Ilyas Vohra, Kamran Sadiq, Mubashir Iqbal, Ayaz-ur- Rehman

**Affiliations:** Department of Paediatrics and Child Health, Aga Khan University Hospital, Karachi, Pakistan

**Keywords:** Paediatrics, Dysphagia, Plummer–Vinson, Endoscopy

## Abstract

**Background:**

Plummer–Vinson syndrome (PVS) is characterized by a triad of symptoms consisting of microcytic hypochromic anaemia, oesophageal webs, and dysphagia. PVS is commonly found in women in the fourth and fifth decades of life and is rarely reported in the paediatric population.

**Case presentation:**

We report the case of a 1-year-old male South Asian child who presented with dysphagia and anaemia for 4 months and frequent episodes of vomiting after ingesting semisolid and solid food. A complete blood analysis revealed microcytic hypochromic anaemia. An oesophagogram revealed circumferential narrowing of the upper thoracic oesophagus. Based on these findings, our suspicion was that the patient had an oesophageal web and vascular ring. Oesophageal dilation was performed with a Savary-Gilliard dilator; initially, 5 mm and 7 mm probes were used, and final dilation with a 9 mm probe was performed.

**Conclusion:**

Although rare in paediatric patients, a high suspicion of this syndrome is necessary in these patients to provide relief to the patient for better growth and development. Iron supplements increase the haemoglobin level but do not subside dysphagia, and oesophageal dilation is needed to open the blocked enteral pathway.

## Background

Plummer–Vinson syndrome (PVS) is characterized by a triad of symptoms consisting of microcytic hypochromic anaemia, oesophageal webs, and dysphagia. PVS is commonly found in women in the fourth and fifth decades of life [1] and is rarely reported in the paediatric population.

An oesophageal web is a thin (2–3 mm), eccentric, smooth extension of normal oesophageal tissue consisting of mucosa and submucosa that can occur anywhere along the length of the esophagus but is typically located in the anterior post cricoid area of the proximal esophagus. The pathogenesis of this syndrome remains unclear, and iron deficiency is the most accepted etiology. Herein, we present a case of a 1-year-old male patient who presented with classic symptoms of PVS.

## Case presentation

We present the case of a 1-year-old boy who was brought to our attention due to a 4-month history of difficulty swallowing solid foods and frequent episodes of vomiting following ingestion of semisolid and solid foods. Despite seeking treatment from multiple general physicians, the symptoms did not abate.

Upon presentation, the patient exhibited a weight-for-length percentile of 87 and a Z score of 1.15. He appeared pale but was otherwise stable, with no signs of hepatosplenomegaly. On laboratory investigation, a peripheral smear revealed microcytic hypochromic anaemia, which was also a consistent finding in his previous laboratory, and elevated red cell distribution width (RDW) and low ferritin levels [Table 1] supported the diagnosis of iron deficiency anaemia.

Although there was no history of atopy, we conducted food allergy tests and IgE assessments, all of which returned normal results, ruling out allergic causes.

**Table 1:** Biochemical investigations

Hemoglobin	Hematocrit	MCV	RBC	TLC	Platelets	RDW	Ferritin
***8.8***	***30***	***60***	***4.94***	***11.5***	***341***	***22.5%***	***5.45***

**Fig. 1:**
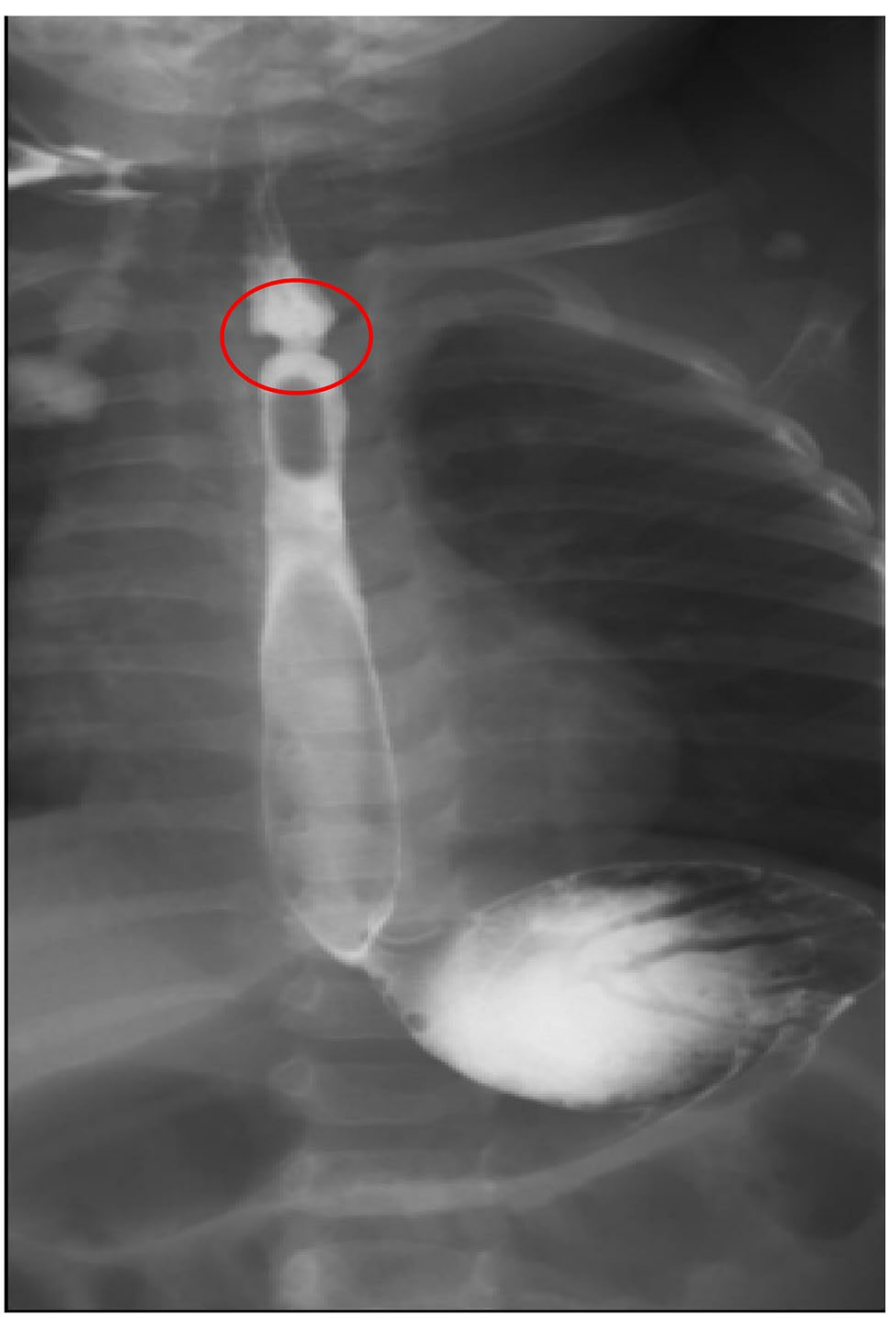
Esophagogram showing esophageal constriction

**Fig. 2:**
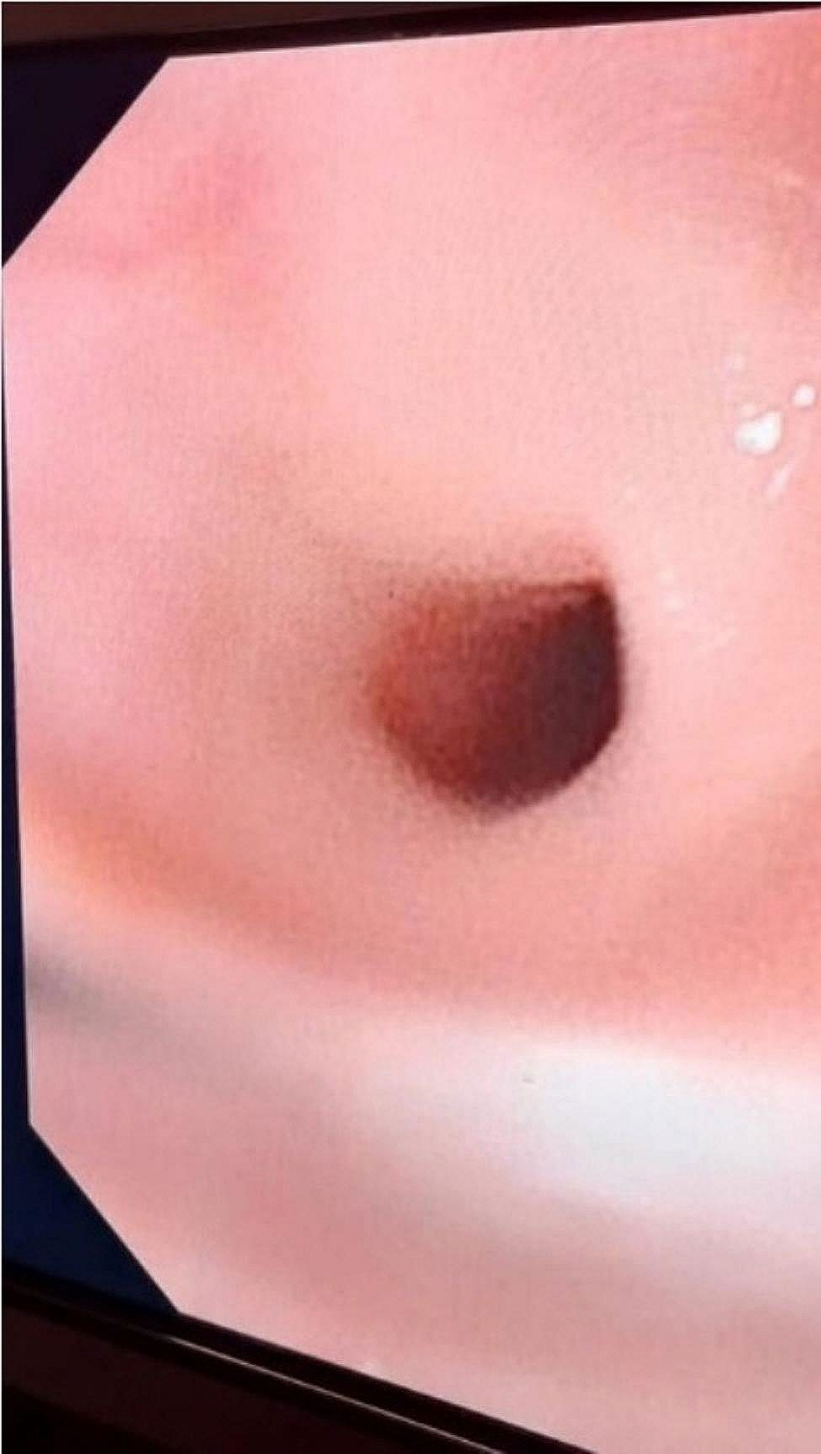
Predilation endoscopic image

An esophagogram revealed circumferential narrowing of the upper thoracic esophagus with a jet phenomenon, mild proximal dilatation, and contrast hold-up [Fig 1]. The rest of the esophagus showed normal motility and caliber. No other filling defects or abnormal extrinsic impressions were observed. Endoscopy confirmed the presence of an oesophageal web in the proximal esophagus [Fig 2]. Our initial suspicion was an oesophageal web and vascular ring due to the patient’s dysphagia and suggestive esophagogram findings. However, financial constraints prevented us from performing a contrast CT scan to confirm the vascular ring. Therefore, we proceeded with treatment for an oesophageal web based on the esophagography findings.

**Fig. 3:**
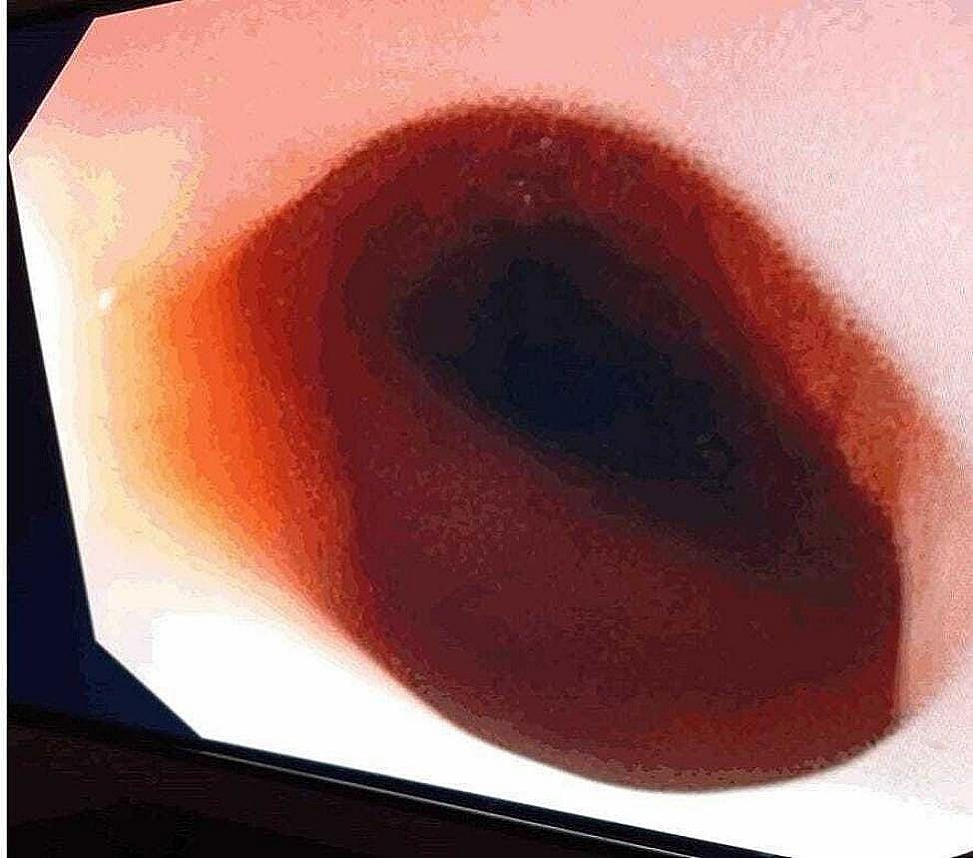
Postdilation endoscopic image

During endoscopy, we identified an oesophageal web in the proximal esophagus, characterized by a membrane protruding into the lumen. We performed oesophageal dilation using a Savary-Gilliard dilator, gradually increasing from a 5 mm to 9 mm probe [Fig. 3].

The patient remained hemodynamically stable post procedure, and his recovery was uneventful. Solid food reintroduction was successful, and the patient was discharged with oral iron supplements. Due to the patient’s remote residence, follow-up was conducted via phone, revealing no recurrence of symptoms after three weeks, with the patient tolerating both liquid and solid diets well. A clinic visit is scheduled for a three-month follow-up.

## Discussion

Plummer–Vinson syndrome (PVS) is typically a combination of iron deficiency anaemia (IDA), difficulty swallowing and oesophageal web [2]. This disease was named after Henry Stanley Plummer and Porter Paisley Vinson, who, in 1912, reported a series of patients with chronic iron deficiency anaemia, dysphagia and constriction of the upper oesophagus without anatomic stenosis; however, Paterson DR and Brown-Kelly A were the first to describe the classic clinical features of the syndrome [3]. The most common sign of an oesophageal web is difficulty swallowing solid foods. Dysphagia is usually progressive and involves solids rather than liquids. Frequent choking, recurrent vomiting, and food blocking have also been reported [4]. Anaemia causing pallor, fatigue and weakness are additional features in the clinical picture of PVS. The pathogenesis of the syndrome is still vague, but as per the available data, iron deficiency is the most likely mechanism [5]. The other possibility is that treatment for oesophageal stricture may lead to improvement in IDA, which suggests a more complex relationship between these conditions. This finding supports the concept that an oesophageal web or stricture is the primary cause of PVS, with IDA being a secondary consequence. However, further research is needed to fully understand the complex interplay between IDA and PVS.

Dysphagia requires radiological assessment, a non-invasive modality, and barium swallow is of utmost importance in revealing the cause of oesophageal lumen obstruction. It can identify conditions such as oesophageal webs, rings, strictures, tumours, and extraesophageal compression [6].

A meta-analysis conducted in France highlighted that all children with PVS experienced iron deficiency anaemia, and no immunological disease was reported. Compared to that in adults, endoscopic dilation is usually necessary because dysphagia patients are less susceptible to iron supplementation. A single dilation was usually sufficient [7]. Different types of dilators are available. Fixed diameter push-type dilators such as flexible Savary–Giliard bougies and radial expanding hydropneumatic dilators that pass over a guide wire or through the channel of the endoscope. With regard to dilators, there is no established global standard, and the use of dilators depends on individual expertise and available resources. A meta-analysis conducted in 2018 showed better efficacy with balloon dilation and less risk of perforation due to the uniform radial force [8].

## Conclusion

Although Plummer–Vinson syndrome is rare in the paediatric population, it should be considered in patients with dysphagia and iron deficiency anaemia. The prevalence of paediatric PVS is similar to that of the adult population, and multidisciplinary team meetings are vital when dealing with such cases; moreover, oesophageal dilation is important for untangling the oesophageal web.

## Data Availability

The dataset used during the current study is available from the corresponding author upon reasonable request.

## Declarations

### Patient’s perspective

My child started to have this issue of vomiting for 4 months. We have visited multiple doctors, and they ordered multiple blood tests, but nothing was useful. Most of them prescribed just multivitamins. We have travelled here for the sake of our child; we want him to be better like other children. We will perform this endoscopy, but we are still concerned about recurrence and the options we are left with if this recurs.

This perspective was. given by the father and translated by the author.

## Abbreviations

PVS: Plummer–Vinson syndrome
IDA: Iron Deficiency Anaemia
